# Effect of Biosilicate^®^ Addition on Physical–Mechanical and Biological Properties of Dental Glass Ionomer Cements

**DOI:** 10.3390/jfb14060302

**Published:** 2023-05-30

**Authors:** Gabriela de Alencar Pinto Magalhães, Joshua J. Thomson, Cristine Smoczer, Laura Ann Young, Adaias O. Matos, Rafael Rocha Pacheco, Maria Trevelin Souza, Edgar Dutra Zanotto, Regina Maria Puppin Rontani

**Affiliations:** 1 Department of Health Sciences and Pediatric Dentistry, Pediatric Division, Piracicaba Dental School, UNICAMP, State University of Campinas, Piracicaba 13414-903, Brazil; gabrielaapmag@gmail.com; 2 Division of Integrated Biomedical Sciences, University of Detroit Mercy School of Dentistry, Detroit, MI 48208, USA; 3 Division of Clinical Essentials and Simulation, University of Detroit Mercy School of Dentistry, Detroit, MI 48208, USA; 4 Department of Restorative Sciences, Dental College of Georgia at Augusta University, Augusta, GA 30912, USA; 5 Vitreous Materials Laboratory, Department of Materials Engineering, Center for Research, Education and Technology in Vitreous Materials (CeRTEV), Federal University of São Carlos (UFSCar), São Carlos 13565-905, Brazil

**Keywords:** glass ceramic, Biosilicate^®^, glass ionomer cement, ion release, bioactivity, antimicrobial, biocompatibility

## Abstract

This study investigated the influence of incorporating Biosilicate^®^ on the physico-mechanical and biological properties of glass ionomer cement (GIC). This bioactive glass ceramic (23.75% Na_2_O, 23.75% CaO, 48.5% SiO_2_, and 4% P_2_O_5_) was incorporated by weight (5%, 10%, or 15%) into commercially available GICs (Maxxion R and Fuji IX GP). Surface characterization was made by SEM (*n* = 3), EDS (*n* = 3), and FTIR (*n* = 1). The setting and working (S/W time) times (*n* = 3) and compressive strength (CS) were analyzed (*n* = 10) according to ISO 9917-1:2007. The ion release (n = 6) was determined and quantified by ICP OES and by UV-Vis for Ca, Na, Al, Si, P, and F. To verify cell cytotoxicity, stem cells from the apical papilla (SCAP) were exposed to eluates (*n* = 3, at a ratio of 1.8 cm^2^/mL) and analyzed 24 h post-exposure. Antimicrobial activity against *Streptococcus mutans* (ATCC 25175, NCTC 10449) was analyzed by direct contact for 2 h (*n* = 5). The data were submitted for normality and lognormality testing. One-way ANOVA and Tukey’s test were applied for the working and setting time, compressive strength, and ion release data. Data from cytotoxicity and antimicrobial activity were submitted for Kruskal–Wallis’ testing and Dunn’s post hoc test (α = 0.05). Among all experimental groups, only those with 5% (wt) of Biosilicate^®^ showed better surface quality. Only M5% showed a comparable W/S time to the original material (*p* = 0.7254 and *p* = 0.5912). CS was maintained for all Maxxion R groups (*p* > 0.0001) and declined for Fuji IX experimental groups (*p* < 0.0001). The Na, Si, P, and F ions released were significantly increased for all Maxxion R and Fuji IX groups (*p* < 0.0001). Cytotoxicity was increased only for Maxxion R with 5% and 10% of Biosilicate^®^. A higher inhibition of *S. mutans* growth was observed for Maxxion R with 5% of Biosilicate^®^ (less than 100 CFU/mL), followed by Maxxion R with 10% of Biosilicate^®^ (*p* = 0.0053) and Maxxion R without the glass ceramic (*p* = 0.0093). Maxxion R and Fuji IX presented different behaviors regarding Biosilicate^®^ incorporation. The impacts on physico-mechanical and biological properties were different depending on the GIC, but therapeutic ion release was increased for both materials.

## 1. Introduction

Glass ionomer cement (GIC) is commonly employed in minimally invasive dental procedures, such as atraumatic restorative treatment (ART) [1,2], due to its durability and good clinical performance over time [3]. GIC possesses advantageous properties such as fluoride release, chemical adhesion to teeth, biocompatibility, and a thermal expansion coefficient similar to dentine [4]. The standard setting reaction of glass ionomer cement occurs when an aqueous polymeric acid solution is neutralized by a glass powder [5]. Aluminum, calcium, fluoride, sodium, and phosphate cations are leached from the glass surface in the first minute of the reaction, leading to the formation of ionic complexes [6]. The ionic complex formations yield calcium polyacrylate or strontium polyacrylate, followed by aluminum polyacrylate, which raises the cement’s viscosity [6,7]. Ion loss affects the glass structure, and acid attacks Si near the surface, forming silica gel [6]. Partially reacted glass particles act as reinforcing fillers in the polymeric matrix. The duration of the setting reaction is commonly within the range of 2 to 6 min [8].

The fluoride release of these materials is inadequate to promote remineralization of caries-affected dentin after an ART procedure [4], despite its ability to inhibit plaque formation and provide an anti-cariogenic effect [9,10,11,12]. GICs have undergone material improvements since their development, particularly in the glass particle and polyacid components [4,13,14]. GIC has been modified with bioactive materials, including hydroxyapatite, chitosan, bioglasses, and even processed bovine dentin [15]. Bioactive materials have been researched for the purpose of creating a restorative cement that can effectively treat challenging cavities, such as those found in deep caries lesions and cervical lesions. This material should possess remineralizing properties, antimicrobial activity, and chemical interactions with dental tissues [16,17].

The incorporation of bioactive glass (BAG) into glass ionomer cements has been shown to induce the remineralization of dentin [18,19,20,21]. Dr. Larry Hench invented BAG in 1969 [22]. Briefly, when BAG is immersed in an aqueous solution (i.e., artificial saliva or simulated body fluid), surface hydrolysis leads to a rapid cation exchange of sodium, calcium, and phosphate with H+ ions from the solution. As the acidity of the solution gradually increases, soluble silica is lost to the solution as Si(OH)_4_ and condenses into a silica-rich layer. Ca^2+^ and PO_4_^3−^ migrate back to the surface of that silica-rich layer, forming an amorphous calcium phosphate layer that crystallizes to form hydroxyapatite.

Bioactive glasses exhibit notable bioactivity, but their mechanical properties are low [23]. Thus, bioactive glass ceramics were developed to improve their mechanical properties. However, as their crystalline volume increases, the rate of apatite formation in vitro decreases significantly. The crystallization of these glasses renders them inert materials. Biosilicate^®^ is a bioactive glass ceramic that achieves high crystallinity and mechanical properties through controlled crystallization while maintaining its bioactivity [24]. Biosilicate^®^ is a bioactive glass ceramic composed of 23.75% Na_2_O, 23.75% CaO, 48.5% SiO_2_, and 4% P_2_O_5_. It is available in two forms: BS-1P, which has one crystalline phase of sodium calcium silicate (Na_2_CaSi_2_O_6_), and BS-2P, which has two crystalline phases, Na_2_CaSi_2_O_6_ and sodium calcium phosphate (NaCaPO_4_) [25].

This material is highly bioactive, osteoconductive, osteoinductive, non-cytotoxic, and non-genotoxic, and it shows antibacterial properties [25]. Biosilicate^®^ has been effectively utilized in the medical and dental fields for over 20 years. Its applications include treating dentine hypersensitivity [26], serving as an enamel remineralization agent [27], and functioning as an antimicrobial agent in intracanal pastes [28]. It may be feasible to enhance the bioactivity and therapeutic ion release of GICs while maintaining their mechanical properties.

This study aims to evaluate the effects of Biosilicate^®^ on the setting behavior, mechanical properties, ion release, cytotoxicity, and antimicrobial activity against *S. mutans* of commercially available conventional and high-viscosity GICs. Thus, the research hypotheses tested are: (I) Biosilicate^®^ incorporation into GIC will affect their setting behavior and mechanical properties; (II) Biosilicate^®^ incorporation into GICs increases their ion release, (III) biocompatibility, (IV) and antimicrobial activity.

## 2. Materials and Methods

### 2.1. Formulation of Experimental Restorative Materials and Group Assignments

Biosilicate^®^ microparticles (D50 = 5 μm) were synthesized by melting followed by heat treatment [29]. Two glass ionomer cements were investigated: [M] Maxxion R (FGM, Joinville, Santa Catarina, Brazil) and Fuji IX GP (GC Corporation, Tokyo, Japan). Table 1 shows the manufacturers and compositions of the materials used in the study. Biosilicate^®^ and the powder portions of each GIC were weighed using a 0.1 mg analytical balance (Chyo Balance JK 180; Chyo Corp., Tokyo, Japan). Then, Biosilicate^®^ was manually incorporated into Maxxion R and Fuji IX powders in concentrations of 5, 10, or 15% wt. Table 2 shows the group distributions, formulations, and powder/liquid (P/L) ratios used in this study. Maxxion R (M0%) and Fuji IX (F0%) without Biosilicate^®^ were used as control groups. M0% and F0% were manipulated following each GIC manufacturer’s instructions. For experimental groups F5%, F10%, and F15%, the P/L ratio was 1.8/1 (wt/wt) to improve handling conditions (when touched or drawn apart, these cements formed strings/fibrils that prevented proper handling). The P/L ratio for Maxxion R groups was kept at 1.5/1 (wt/wt).

### 2.2. Scanning Electronic Microscopy (SEM) and Energy-Dispersive X-ray Spectroscopy (EDS)

The powders of each material (Fuji IX GP, Maxxion R, and Biosilicate^®^) were individually applied onto a carbon tape mounted on plastic stubs using a plastic rod and sputter-coated with gold (MED 010, Balzers, Liechtenstein) [30] to evaluate particle morphology. In addition, cylindrical molds (4.0 mm in diameter and 3.0 mm in height) were fabricated. The cements were manipulated, inserted into the molds (*n* = 3), and stored for 24 h in relative humidity (100%). Following this, the cylindrical specimens were split off using a chisel and a hammer, finished and polished using #320 and #400 sandpaper with 99° ethanol, and stored for 24 h in a desiccator (Pyrex, São Paulo, SP, Brazil) at 37 °C. The specimens were mounted on the stubs, as previously described. A scanning electron microscope was used (JSM-5600LV; Jeol, Tokyo Japan) and images were taken at magnifications of 1000×, 5000×, operating at 15 kV with a ray width of 25–30 nm and working distance of 10–15 mm. Energy-dispersive X-ray spectroscopy (EDS) was performed to qualitatively estimate the number of ions (Ca, P, Si, Al, Na, F) on the cross-section (as previously described) of the cylindrical specimens (*n* = 3). The specimens were fixed on carbon tape over a plastic stub, coated with carbon (MED 010, Baltec), and analyzed using 15 kV with a working distance of 10 mm, spot size of 25 mm, and ×500 magnification. The images obtained in the SEM were used for qualitative analysis of the GIC surfaces, as were the spectra obtained by the EDS evaluation.

### 2.3. Fourier Transform Infrared Spectroscopy (FTIR)

To investigate GIC setting dynamics, FTIR spectra were collected over time (*n* = 1). The cements were mixed, immediately placed and kept in the spectrometer, and curves were obtained after 1, 5, and 60 min [31]. The analysis was performed with a spectral resolution of 4 cm^−1^ in the region of 4000 to 400 cm^−1^ (Perkin Elmer, Spectrum GX. DE), though only the spectral range of interest (1800 to 900 cm^−1^) was represented. FTIR spectra were baseline-normalized using a specific software (Spectragryph Software, Germany, version 2.16.1) and plotted in statistical software (GraphPad, Prism, version 9.5.0). The peaks of interest were searched for in the range of 1700 to 1715 cm^−1^ for poly-carboxylic acid and 1400 to 1640 cm^−1^ for poly-carboxylate salts [31,32,33,34].

### 2.4. Working and Setting Time

The working and setting times of evaluated GICs were measured by using Gilmore needles in triplicate [35,36]. The cements were mixed and placed in metallic molds of 8.00 mm × 10.00 mm × 5.00 mm on top of an aluminum foil. A large-diameter needle (2.12 mm) with a weight of 113 g was applied, and the working time was determined as soon as the needle no longer indented the surface. Then, a small-diameter needle (1.06 mm) with 453.6 g of mass was positioned perpendicularly to the cement surface for 5 s. The indentations were repeated every 30 s in different spots until the needle failed to make a completely circular indentation, determining the setting time.

### 2.5. Compressive Strength (CS)

Eighty (80) cylindrical specimens (*n* = 10) were prepared according to ISO 9917-1:2007 using custom silicone molds (4.00 mm in diameter and 6.00 mm in height). Cements were mixed and placed into molds between two glass slides protected by a transparent polyester film (Mylar^®^). Specimens were removed from the molds after 30 min and stored at 100% relative humidity in an incubator at 37 °C for 24 h. The experiments were conducted using a universal testing machine (Model 5544, Instron, Canton, MA, USA) with a load of 50 N/min and a speed of 0.75 mm/min. The compressive strength (CS) was calculated (MPa) according to the formula:

$$Cs=\frac{4p}{\pi .D^{2}}$$

where *p* represents the load (Newton) exerted by the load cell until the sample failure, and *D* is the diameter of each specimen (mm), measured using a digital caliper (Mitutoyo, Japan).

### 2.6. Ion Release by Inductively Coupled Plasma Optical Emission Spectrometry (ICP OES) and Ultraviolet–Visible Spectrometry (UV-Vis)

Forty-eight (48) cylindrical specimens (*n* = 6) were made as previously described for CS and stored in an incubator at 37  °C for 1 h. The specimens were removed from the molds and placed in 20 mL of deionized water (DI) in plastic tubes (surface area/mL, 1 cm^2^/mL) [37]. The determination of Ca, Na, Al, Si, P, and F ions in eluates was made using inductively coupled plasma optical emission spectrometry (ICP OES), using iCAP 6000 (Thermo Fischer Scientific, Madison, WI, USA) and ultraviolet–visible spectrometry (UV-Vis). Quantifying and detection limits were, respectively, 0.050 and 0.015 ppm for Ca, Na, Al, Si, and P ions. Ultraviolet–visible spectrometry (UV-Vis) (DR/2500, HACH, Ames, Iowa, USA) was used to determine F ion release according to Standard Methods for the Examination of Water and Wastewater (SMWW), 4500-F-D.-SPANDNS. To quantify and detect F ions, the limits considered were 0.100 and 0.030 ppm, respectively. To calculate the cumulative concentration of the respective ions, the limit values of quantification and detection of the analysis methods used were normalized.

### 2.7. GIC Cytotoxicity

The cytotoxicity was measured as part of the biocompatibility assessment. Twenty-four specimens (*n* = 3) of 5.00 mm in diameter and 1.00 mm in thickness were made using polyvinylsiloxane (PVS) molds and allowed to set for 1 h at 37 °C. Subsequently, specimens were added to 300 μL of cell culture media for 24 h at 37 °C (surface area/mL was 1.8 cm^2^/mL) [38]. The study used stem cells from Apical Papilla (hSCAPs), which were donated by the University of Texas Health Science Center Dental School, San Antonio, TX, USA. These cells share common features with dental pulp stem cells (hDPSC) [39] and exhibit similar cell viability and proliferation under the same culture conditions [40,41]. Thus, the Promega CellTox^TM^ Green assay was performed to assess the cytotoxicity of GICs on hSCAPs (RP-89 cell line). Cells were seeded at 10,000 cells/well in a black opaque-walled 96-well plate and maintained in α-MEM (Gibco, Grand Island, NY, USA) supplemented with 10% fetal bovine serum (Gemini, West Sacramento, CA, USA), 1% L-glutamine, and 1% penicillin/streptomycin (Sigma-Aldrich, St. Louis, MO, USA) at 37 °C and 5% CO_2_. After 24 h, the cell culture media were replaced by GIC-conditioned media. Cytotoxicity was evaluated using the Promega CellTox Green^®^ assay at 24 h post-exposure, as per the manufacturer protocol. Fluorescence was read on a Spark^®^ multimode microplate reader (Tecan, Männedorf, Switzerland) with excitation/emission wavelengths of 485 nm/520 nm. Cells exposed to the provided lysis buffer were considered positive controls, while cells incubated in cell culture media alone were negative controls. Data were reported as percentage of dead cells in a population, relative to the positive control set arbitrarily to 100%.

### 2.8. Antimicrobial Activity: Direct Contact with Disk Surfaces

Forty specimens (*n* = 5) of 15.00 mm in diameter and 1.00 mm in thickness were made using custom PVS molds. After 1 h setting at 37 °C, each specimen was placed into a well of a 6-well plate and sterilized under UV light for 20 min per side. *Streptococcus mutans Clarke* (ATCC 25175, NCTC 10449) was inoculated in BHI broth (BD Difco^TM^, Becton, Dickinson, and Company, Sparks, MD, USA) and incubated at 35 ± 2 °C supplemented with 5% CO_2_ for 24 h. Then, the overnight inoculum was centrifuged and resuspended to an optical density of 600 nm (OD_600_) = 1.0, approximately 1 × 10^9^ colony-forming units/mL (CFU/mL) in dilute BHI (1:500 BHI in sterile DI water). Bacterial suspension was diluted 100-fold, and 50 μL of the diluted bacterial suspension was added to each specimen surface (approximately 5.0 × 10^5^ CFU/specimen). Then, a UV-sterilized plastic coverslip (15.00 mm in diameter) was aseptically placed onto each disk and incubated at 100% relative humidity at 25 ± 2 °C. After 2 h of direct contact, 950 μL of sterile phosphate-buffered saline (PBS) was added to each specimen/well, and the plate was placed on an orbital shaker (Orbit^TM^ 1000, Labnet International Inc., Edison, NJ, USA) at 150 rpm for 2 min to remove bacteria from the surface of the specimen. Then, liquid from each well (1 mL) was collected and serially diluted for plating onto BHI agar. Plates were incubated at 35 ± 2 °C + 5% CO_2_ for 48 h. The colonies formed were counted using a colony counter (Bantex^®^ 920A, American Bantex Corp, Burlingame, CA, USA) and CFU remaining after surface exposure was calculated.

### 2.9. Data Analysis

Statistical analysis was performed using GraphPad Prism 9 version 9.5.0 (November 2022). The Shapiro–Wilk test was utilized to examine the normality and lognormality of cell viability data with a significance level of 0.05. The compressive strength and ion release presented normal distribution, and the data were submitted to one-way ANOVA followed by a Tukey’s multiple comparisons test. Due to the non-normal distribution of cell viability and antimicrobial data (*p* < 0.05), Kruskal–Wallis’ test was recommended to detect differences in group medians (*p* < 0.05). The multiple comparison post hoc test developed by Dunn was used to identify differences between experimental groups.

## 3. Results

### 3.1. SEM and EDS Analyses

**Figure 1:**
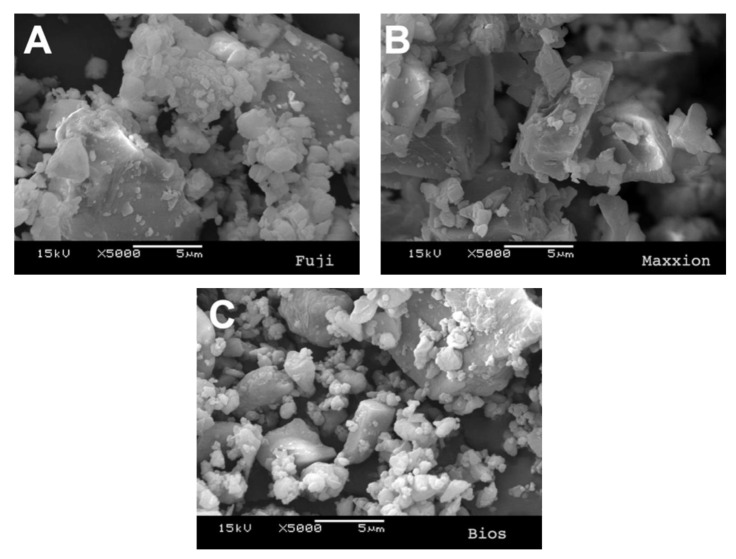
SEM analysis of GIC and Biosilicate^®^ powders. (**A**) Fuji IX GP, (**B**) Maxxion R, and (**C**) Biosilicate^®^.

Figure 1 depicts the particle shape and size of Fuji IX (Figure 1A), Maxxion (Figure 1B), and Biosilicate^®^ (Figure 1C). All materials were observed to have non-uniform particle sizes. As seen in SEM images, Fuji IX particles are more sphere-like (octahedron shape), whereas Maxxion R particles have sharp edges (hexahedron shape). Small Biosilicate^®^ particles have a sphere-like form and are clustered on large particles (hexahedron shape), indicating a greater particle size distribution. The particles were categorized based on their shapes [42].

**Figure 2:**
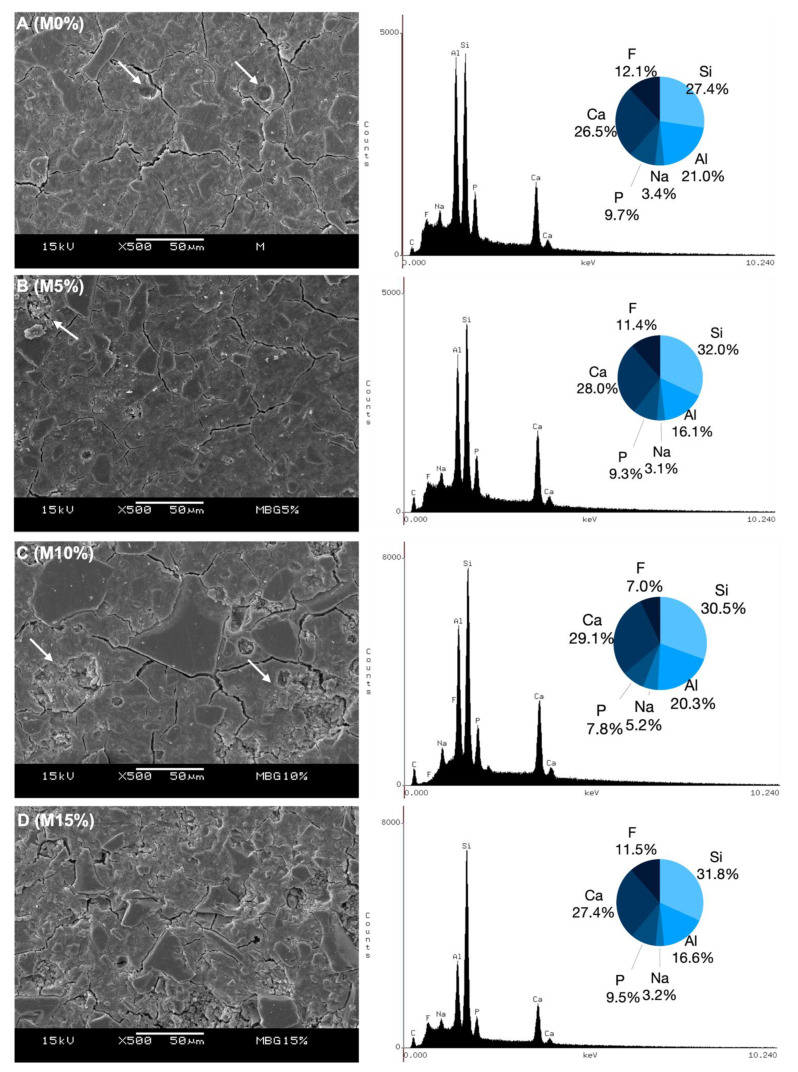
Representative SEM micrographs and EDS histogram of the main ions present on their internal surface (%wt) as determined by EDS. On M0% (**A**) white arrows indicate presence of pores. Unreacted small particle clustering is shown in (**B**,**C**), indicated by white arrows at M5% and M10%, respectively. These clusters increased with higher concentrations of Biosilicate^®^, as observed in (**D**) at M15%.

**Figure 3:**
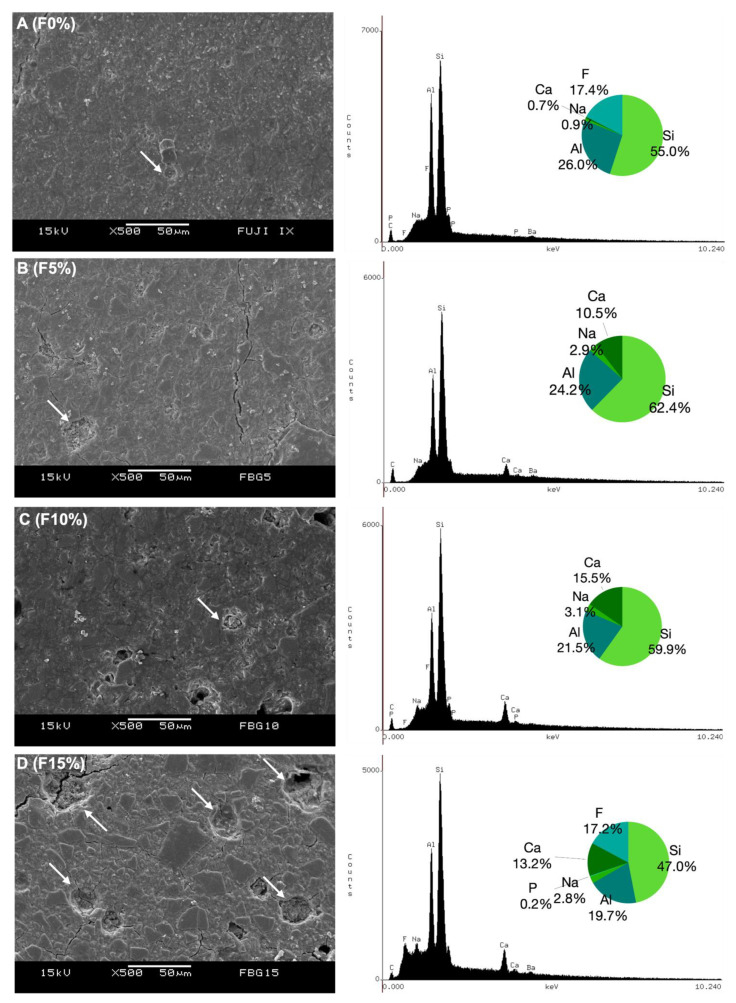
Representative SEM micrographs from Fuji IX groups and EDS histogram of the main ions present on their internal surface (%wt) as determined by EDS. The white arrow in (**A**) indicates the presence of a pore on F0%. (**B**–**D**) point out unreacted small particle clustering at F5%, F10%, and F15%, respectively, as indicated by white arrows. The observed increase in clusters was associated with higher concentrations of Biosilicate^®^, which was observed in F15%.

Figure 2 and Figure 3 depict the surface morphology of the specimens. All groups presented crack lines on their specimens’ surfaces. However, Fuji IX groups were observed to have smoother surfaces with less apparent porosity and fewer cracks than Maxxion R groups. All experimental cements exhibited clusters of unreacted small particles whose number increased with increasing Biosilicate^®^ concentrations (white arrows). Figure 2 also shows histograms and the EDS spectra (%wt): calcium (Ca), phosphorus (P), silicon (Si), aluminum (Al), sodium (Na), and fluorine (F) in the final mixture for each cement.

### 3.2. FTIR

**Figure 4:**
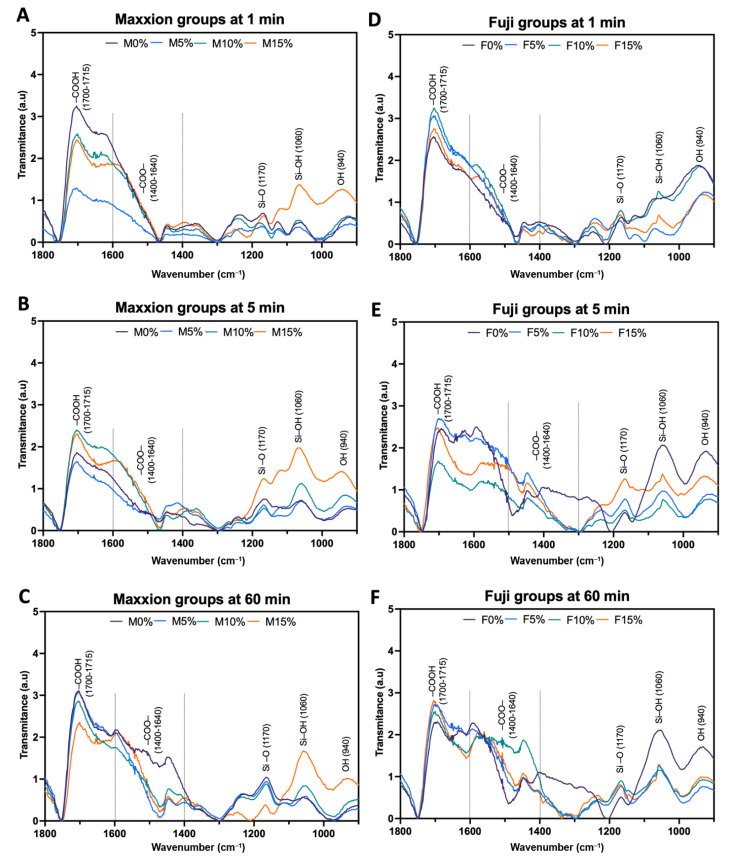
Data from the FTIR of Maxxion R (**A**–**C**) and Fuji IX groups (**D**–**F**) at 1, 5, and 60 min.

Figure 4 depicts the FTIR spectrum of cements at 1, 5, and 60 min after initial mixing. Data from the FTIR of all samples showed absorption bands in 1715, 1560, 1455, 1415, 1170, 1060, and 940 cm^−1^. As the materials set, the intensity of the –COOH groups of polyacrylic acid (1715 cm^−1^) decreases and reorganizes as COO- (polycarboxylate salts) groups (1400–1640 cm^−1^), as indicated by the dashed lines. The loading graph reveals that these two bands vary significantly at 1, 5, and 60 min of setting. The neutralization reaction appeared at 1170 (Si-O), 940 (O–H) cm^−1^, and with the silica gel (Si-OH) at 1060 cm^−1^.

### 3.3. Setting and Working Time

**Figure 5:**
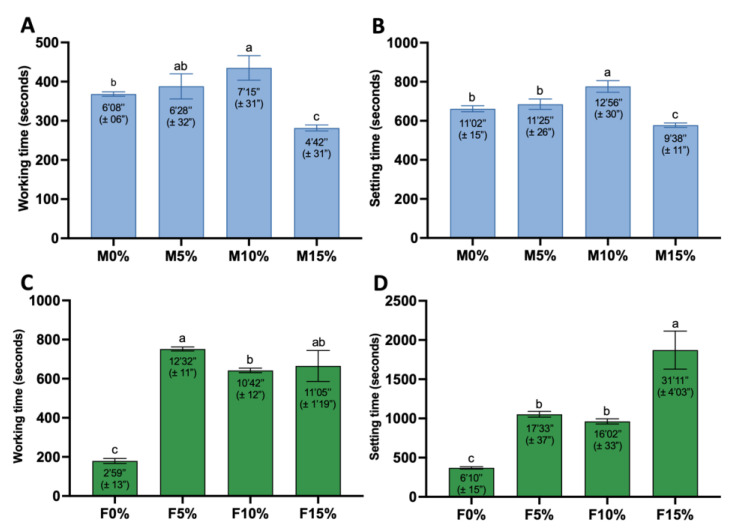
Working and setting times of Maxxion groups (**A**,**B**) and Fuji IX groups (**C**,**D**) respectively. Error bars represent standard deviations. One-way ANOVA and Tukey’s, performed separately for working and setting time. Different letters indicate significant differences between mean values of working and setting times (*p* < 0.0001).

Figure 5 shows the working and setting times for cements. M10% exhibited the longest working time for Maxxion R (Figure 4A,B), whereas M15% exhibited the shortest. M0% and M5% showed similar working and setting times, and no significant differences were found (*p* = 0.7254 and *p* = 0.5912). There was no significant difference in working time between M5% and M10% (*p* = 0.1314). M15% working and setting times were significantly shorter than M0% (*p* = 0.0072 and *p* = 0.0071). All experimental groups for Fuji IX (Figure 4C,D) had significantly longer working and setting times than F0%. F5% had the longest working time, followed by F15% and F10%, while F0% had the shortest. F15% did not differ significantly from F5% (*p* = 0.1150) and F10% (*p* = 0.9017) regarding working time. However, F15% had the shortest setting time, followed by F5% and F10%, which were similar (*p* = 0.8043).

### 3.4. Compressive Strength (CS)

**Figure 6:**
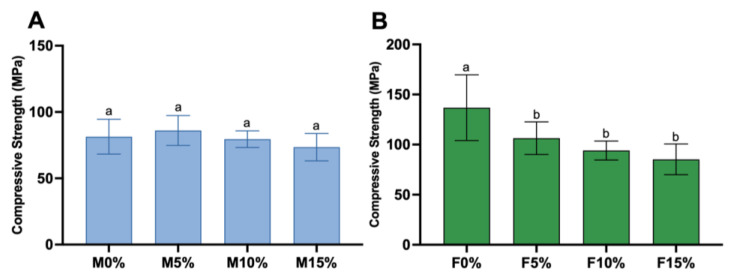
Compressive strengths of GICs. (**A**) Maxxion without Biosilicate^®^ (M0%), M5%, M10%, and M15%. (**B**) Fuji IX without Biosilicate^®^ (F0%), F5%, F10%, and F15%. Error bars represent standard deviations. Different lowercase letters indicate significant differences among mean values of compressive strength (MPa) in groups (*p* < 0.0001).

Figure 6 depicts the results for compressive strength. The addition of Biosilicate^®^ to Maxxion R (Figure 6A) did not compromise the CS. No significant differences were found between the Maxxion R groups. In contrast, for Fuji IX groups (Figure 6B), the compressive strength decreased by approximately 30% for F5%, 37% for F10%, and 43% for F15% compared to F0% (*p* < 0.0001). The F10% group did not differ from the F5% and F15% groups. However, F5% was significantly higher than F15% (*p* = 0.0126). Fuji IX groups exhibited a higher compressive strength than Maxxion R, with a maximum value of 149 MPa compared to 75 MPa for Maxxion R.

### 3.5. Ion Release

**Figure 7:**
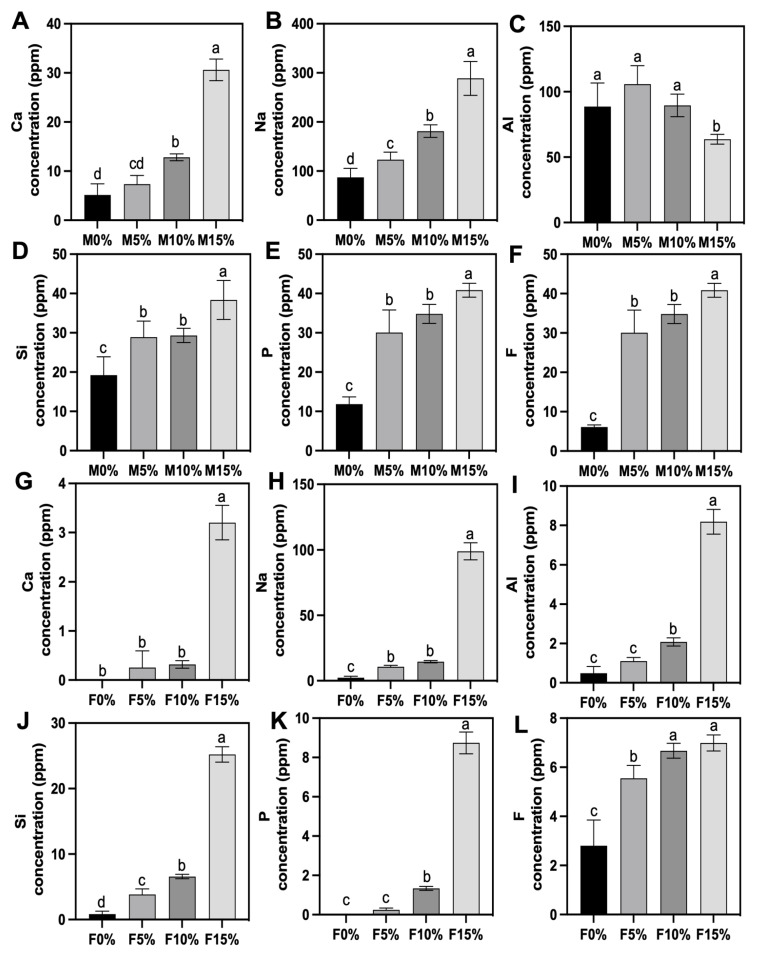
Concentration of ions released in 24 h. Na, Al, Si, P, and Ca determinations on the eluates by ICP OES, and F by UV-Vis. Different letters indicate significant differences in the amount (ppm) of the ion released by each group (Maxxion R: (**A**–**F**); Fuji IX: (**G**–**L**)).

Figure 7 depicts the ions released (ppm) by GICs after 24 h of soaking. In general, for Maxxion R and Fuji IX groups, the higher the concentration of Biosilicate^®^ added, the greater the amount of the studied ions released, except for the Al ion released by Maxxion (3.1.3).

#### 3.5.1. Ca Ions

Compared to Fuji IX groups, Maxxion R (Figure 7A) released a greater amount of Ca ions. M15% released the highest amount of calcium (31 ppm), followed by M10% (12 ppm). M5% and M0% did not differ statistically and released approximately 9 ppm of Ca ions. Group F0% exhibited a release of zero ppm of Ca ions. F5% and F10% did not differ significantly and released a small amount of Ca, approximately 0.5 ppm, as shown in Figure 7G. F15% released a significantly higher amount (3 ppm).

#### 3.5.2. Na Ions

All Maxxion R groups (Figure 7B) differed significantly from one another: M15% (290 ppm) > M10% (180 ppm) > M0% (120 ppm) > M5% (80 ppm). For Fuji IX (Figure 7H), F15% released a higher amount of 100 ppm, followed by F10% and F5%, which were statistically similar (12 ppm) (*p* = 0.2086). F0% released the lowest amount (3 ppm).

#### 3.5.3. Al Ions

M15% released an average of 60 ppm of Al ions, whereas M5%, M0%, and M10% did not differ statistically, releasing approximately 100 ppm (Figure 7C). The highest Al release for Fuji IX groups (Figure 7I) was observed for F15% (8 ppm), followed by F10% (2 ppm). F0% and F5% did not differ statistically and presented the lowest Al release.

#### 3.5.4. Si Ions

M15% released significantly more Si ions than the other Maxxion R groups (38 ppm; see Figure 7D). M5% and M10% released about 28 ppm and were statistically similar (*p* = 0.9981). M0% released the lowest amount (19 ppm). For Fuji IX (Figure 7J), all groups were significantly different from each other (*p* < 0.0001): F15% (25 ppm) > F10% (7 ppm) > F5% (4 ppm) > F0% (1 ppm), with a decreasing amount of Si ions released as the concentration decreased.

#### 3.5.5. P Ions

M15% released the most significant amount of P ions (40 ppm), whereas M0% released the lowest amount (11 ppm). M5% and M10% were not statistically different (approximately 30 ppm) (Figure 7E). No P ions were detected or quantified in F0%. The P ion release by F5%, F10%, and F15% was made possible by incorporating Biosilicate^®^. F5% (0.5 ppm) did not differ statistically from F0% (*p* = 0.4751). F10% (1 ppm) was statistically higher than F0% and F5% (*p* < 0.0001). F15% released the highest amount of P ions among Fuji IX groups (9 ppm) (Figure 7K).

#### 3.5.6. F Ions

The incorporation of Biosilicate^®^ into Maxxion R increased F ion release by a factor of 4 and by a factor of 2.5 for Fuji IX. M15% released the highest amount of F ion (40 ppm), followed by M10% and M5% (35 ppm); the latter two did not differ statistically. M0% presented a significantly lower (5 ppm) F ion release (Figure 7F). Fuji IX by itself (F0%) showed the lowest amount of fluoride release, at 2.5 ppm. F5% released a significantly higher amount (5.7 ppm) compared to F0% (*p* < 0.0001). The highest amount was observed for F15% (7 ppm), which did not differ statistically (*p* = 0.8187) from F10% (6.3 ppm), as shown in Figure 7L.

### 3.6. Cytotocixity

**Figure 8:**
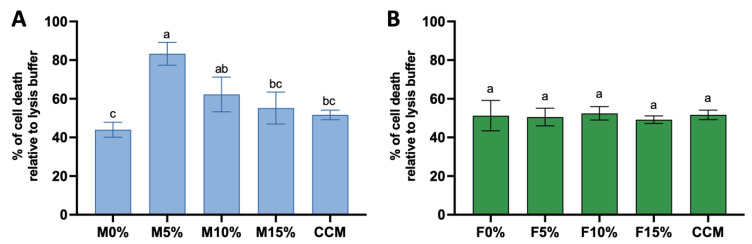
Cytotoxicity at 24 h for Maxxion groups (**A**) and Fuji IX groups (**B**). Percentage of cell death relative to the lysis buffer (100% of death) while the negative control cells were cultured only in α-MEM. Different letters represent statistical differences on the charts.

The percentage of dead cells was calculated relative to the lysis buffer (positive control) set to 100%. M5% presented a significantly higher percentage of dead cells compared to M0% (*p* < 0.0001) and M15% (*p* < 0.0001). M5% appears to be the most cytotoxic since it was the only treatment significantly different from the negative control (*p* = 0.0009). The next most cytotoxic group was M10%, which did not differ from M5% (*p* > 0.9999). Among those Maxxion R groups with Biosilicate^®^, only M15% did not differ from M0% (*p* = 0.0541). A tendency toward decreased cytotoxicity when concentrations above 5% of Biosilicate^®^ have been incorporated was observed. In the Fuji IX groups (Figure 8B), Biosilicate^®^ incorporation did not have an impact on the material cytotoxicity, as the F0%, F5%, F10%, F15%, and CCM groups showed that they were not significantly different (*p* > 0.9999).

### 3.7. Antimicrobial Activity

**Figure 9:**
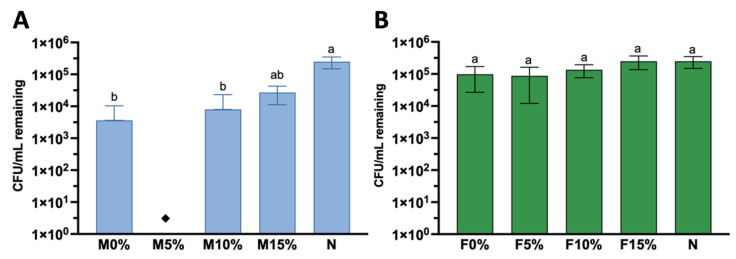
*S. mutans* remaining after 2 h of direct contact with Maxxion groups (**A**) and Fuji groups (**B**). Black diamond (◆) indicates less than 100 CFU/mL recovered (limit of detection). Different letters indicate significant differences among groups (*p* < 0.0001).

The data were analyzed by the Kruskal–Wallis test and Dunn’s multiple comparisons test. After *S. mutans* specimen exposure, a 1-log reduction in surviving bacteria was observed for all Maxxion groups compared to the N group (no specimen, only bacteria). M5% consistently reduced *S. mutans* by 4- to 5-logs compared to all other groups. No colonies (zero) were observed on the plates, meaning that less than 100 CFU/mL remained after direct contact with M5% (indicated by the black diamond in Figure 9A). Therefore, statistical analysis was run comparing the M0%, M10%, M15%, and N groups. Only M0% (*p* = 0.0053) and M10% (*p* = 0.0093) showed statistically reduced CFUs after exposure compared to the N group, although there was no significant difference among M0%, M10%, and M15%. No significant reduction in CFUs was found for all Fuji IX groups when compared to each other or when compared to the N group (Figure 9B).

**Figure 10:**
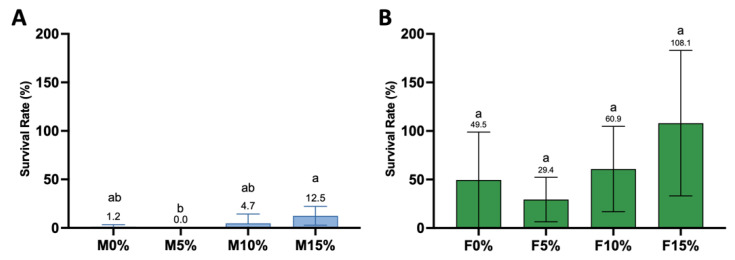
Percentage of survival of *S. mutans* compared to no disk group (only bacteria) for Maxxion groups (**A**) and Fuji groups (**B**). No disk was considered 100% survival. Different letters indicate significant differences among groups.

The percentage of survival of *S. mutans* after 2 h of direct contact was calculated using the N group as a 100% survival reference. Overall, the Maxxion R and Fuji IX groups showed substantial differences in overall *S. mutans* survival. As shown in Figure 10A, M5% presented a 0% (±0.0) survival rate, followed by M0% at 1.2% (±2.7), M10% at 4.7% (±0.8), and M15% at 12.5% (±5.3). M5% consistently differed from M15% (*p* = 0.0046). Regarding Fuji IX groups (Figure 10B), no significant differences were observed in survival rates for F0%, F5%, F10%, and F15% (*p* > 0.0001). Fuji 5% trended towards being the most effective in antimicrobial effects among Fuji IX groups, presenting a 29.4% (±10.7) survival compared to the no specimen group. F0% and F10% presented an intermediate percentage of survival, respectively, 29.4 (±14.9) and 60.9 (±5.3). F15% increased *S. mutans* survival by 8.1% (±5.9), but none of these differences reached statistical significance.

## 4. Discussion

This study aimed to evaluate the effects of adding Biosilicate^®^ (a bioactive glass ceramic composed of 23.75% Na_2_O, 23.75% CaO, 48.5% SiO_2_, and 4% P_2_O_5_) to Maxxion R (conventional) and Fuji IX (high viscosity) glass ionomer cements. The first research hypothesis was accepted, as the incorporation of Biosilicate^®^ in GICs impacted their mechanical properties and setting times. The setting and working times of M5% were found to be comparable to those of M0% in conventional GIC (Maxxion R). Maxxion with 5% Biosilicate^®^ was well tolerated. However, the addition of 10% Biosilicate^®^ resulted in a significant increase in setting time, while the addition of 15% Biosilicate^®^ resulted in shorter setting times. The setting time of GIC with bioglass increased with the increase in the amount of bioglass added [18,19], whereas the opposite trend was observed in the M15% setting behavior. The experimental groups exhibited a significant increase in the working and setting times for the high-viscosity GIC (Fuji IX). Furthermore, the Maxxion R groups did not exhibit any significant variations in compressive strength (CS), while the CS of Fuji IX cements decreased for the experimental groups.

The FTIR spectrum indicated slight structural modifications when comparing the peaks obtained to the conventional glass ionomer cement setting mechanism, as reported by other researchers [34,43,44]. What stands out in the FTIR spectrum is the formation of polysalts and the consumption of polyacrylic acid (–COOH) with time, indicating that there is an interaction between the Biosilicate^®^ and GIC. The polycarboxylate salts in the range of 1400 to 1640 cm^−1^ [33] were also observed in the present study (clearly indicated between dashed lines in Figure 4). The 1409 cm^−1^ range corresponds to calcium carboxylate (-COOCa_2+_), the 1459 cm^−1^ range corresponds to aluminum carboxylate (-COOAl_3+_), and the 1552 cm^−1^ range corresponds to sodium carboxylate (-COONa_2+_) [31,32,34].

The use of GICs with distinct powder and liquid phase compositions may result in diverse effects on the analyzed properties and should therefore be taken into consideration. Fuji IX powder contains fluoroaluminosilicate glass particles and lyophilized PAA. The acid–base reaction is initiated by ionizing PAA in the powder with water. Maxxion powder contains fluoroaluminosilicate and calcium fluoride, while the liquid component consists of polycarboxylic acid, tartaric acid, and water. The inclusion of Biosilicate^®^ in Fuji experimental groups may have impeded the hydration of polyacrylic acid (PAA), leading to a decrease in PAA ionization and a subsequent reduction in the leaching of cationic ions due to an acid attack on the glass. This, in turn, may have resulted in a reduction in the formation of crosslinks within the matrix.

In addition, it has been reported that mixing PAA and glass can facilitate the hydration process, particularly in the presence of unstable glass phases. The dental GIC typically employs the G338 glass system, which consists of three amorphous glass phases: an alumino-silicate (phase 1), a Ca-F-rich (phase 2), and a Ca-F-P-rich (phase 3). The alumino-silicate phase is the most resistant to acid attack by PAA among these phases [45]. When comparing the compositions of Maxxion and Fuji as provided by the manufacturers (Table 1) and the EDS results (Figure 2 and Figure 3), it is clear that Maxxion contains a greater proportion of the components present in phases 2 and 3 and thus reacted more readily. Fuji appears to be more abundant in phase 1 (alumino-silicate), making the reaction more difficult. Depending on the proportion of each glass phase in a GIC, it may be more reactive (as may be the case with Maxxion) or more resistant (as may be the case with Fuji).

For Fuji IX, the P/L ratio was modified from 3.6/1 to 1.8/1 (wt/wt) for enhanced handling conditions. The P/L ratio, polyacid concentration, and powder particle size affect the working and setting times and mechanical properties [15,46]. Since the P/L ratio remained unchanged, the inclusion of Biosilicate^®^ had no impact on the compressive strength of the Maxxion groups. However, the P/L ratio appears to be crucial for the CS of the Fuji IX experimental groups (F5%, F10%, and F15%). A decline in CS was anticipated for these groups.

In addition to differences in the powder compositions of Maxxion R and Fuji IX, it is important to mention that the brand-specific polyacid type can influence the physico-mechanical properties of the GICs [47]. Conventional glass ionomers, such as Maxxion, use less concentrated and less viscous polyacids compared to high-viscosity GICs, such as Fuji IX [8,48]. The mechanical properties are significantly influenced by the concentration and molecular weight of the polycarboxylic acid [13,48]. This may explain why Fuji IX demonstrated superior mechanical properties in comparison to Maxxion, as depicted in Figure 6.

Studies have shown that the inclusion of Al^3+^ in certain bioglass formulations has resulted in an increase in the compressive strength (CS) of glass ionomer cements (GICs) when compared to formulations lacking Al^3+^ [49]. Sodium-containing bioglasses had a negative impact on GICs, while formulations without sodium did not affect their setting or mechanical properties [18,20,50]. Biosilicate^®^ contains sodium oxide (23.75% by wt) and no aluminum. The partial replacement of GIC powder with bioactive glass ceramic resulted in a reduction in the amount of Al^3+^. Aluminum ions (Al^3+^) enhance the strength of the GIC matrix by forming stronger three-dimensional bonds compared to calcium ions (Ca^2+^) [46]. Any imbalance in this process or interference with the glass degradation can have an effect on the cement’s setting and, consequently, its mechanical properties [51].

Bioactive glasses with a high sodium content may release Na^+^ during acid–base reactions, which can negatively affect cement setting reactions, prolong setting time, and compromise mechanical properties [4,49]. In addition, the Al:Si ratio of approximately 1:2 in glass is essential for a satisfactory setting reaction rate, hydrolytic stability, and the ability of glass to form cements [52,53,54]. This study did not directly measure the Al:Si ratio after incorporating Biosilicate^®^ by %wt; thus, an imbalance in the Al:Si ratio should not be discarded in this case.

According to ISO 9917-1:2007, this material must have a minimum working time of 1.5 min and a maximum setting time of 6 min. F0% complied with ISO 9917 in the setting time, while M0% did not meet the requirements. F0% and F5% were the only groups that met the ISO 9917-1:2007 requirement of having CS values greater than 100 MPa. Maxxion groups did not meet this requirement, indicating a need for adjustments to be made to the experimental cements. Testing the reaction between different polyacids and bioglass or glass ceramic-containing GIC could enhance setting behaviors and mechanical properties. The initial reaction in conventional glass ionomer cement involves the neutralization of the aqueous polyacid and glass powder [5]. Calcium (or strontium) polyacrylate is promptly produced by this reaction, while aluminum polyacrylate is formed with a slight delay [7].

The presence of calcium and sodium ions in Biosilicate^®^ may cause charge repulsion, leading to a decrease in the binding of aluminum ions to the polymer chains [13,50]. This can have an impact on the material’s setting behavior and mechanical properties. Polyacrylic acid (PAA) has a lower number of carboxyl groups per repeating unit compared to poly(vinylphosphonic-co-acrylic acid) [55,56], which is utilized as a rate-modifier in some commercial products due to the presence of an additional phosphonic acid group [14]. This allows more protons to attack the glass but also promotes the formation of additional ionic cross-links between metallic cations (e.g., Al^3+^) and the polymeric acid. This results in improved hydrolytic stability and mechanical properties of cement [43].

All experimental groups exhibited crack lines, unreacted particles, and rough surfaces. Small particle clusters of Biosilicate^®^ were also observed in the experimental cements. These concerns are frequently noted in GICs that contain bioglass [21]. Fuji IX groups displayed superior surface smoothness, fewer pores, and fewer cracks compared to Maxxion R groups. The observed variations in these GICs could be attributed to disparities in their composition, particle size, and P/L ratio [57,58]. Lower concentrations (F5% and M5%) of the bioactive glass ceramic induced fewer microstructural disruptions in the GICs.

Higher Biosilicate^®^ concentrations resulted in greater ion release from GICs, thus supporting the acceptance of the second research hypothesis. Overall, the release of Na, Si, P, and F increased with increasing Biosilicate^®^ concentrations. The Ca ion release was significantly higher in Fuji IX, at F15%, and in Maxxion R, at M10% and M15%, respectively. Aluminum release was significantly lower in the M15% group, suggesting that Biosilicate^®^ at this concentration may have reduced the release of aluminum. The descending order of ion release profiles for Maxxion R groups was Na > Al > Si = P = F > Ca. For Fuji IX, the order was Na > Si > Al = P = F > Ca. Maxxion R groups released more ions than Fuji IX groups, as shown in Figure 7.

The Al release range of Maxxion R groups (60 to 105 ppm) was significantly higher than that of Fuji IX groups (<1 to 8 ppm), likely due to the distinct compositions of the two cements. Specifically, Maxxion R contains Ca_2_F and NaF, while Fuji IX does not. Larger cations (e.g., Ca and Na) have been found to disrupt the glass network more efficiently, leading to a reduction in oxygen density and an expansion of the glass network. This expansion facilitates the diffusion of smaller cations (e.g., Al) through the glass [59]. Thus, it is hypothesized that the Al in Fuji powder was crosslinked via carboxylate salts and entrapped in the GIC matrix, leading to minimal Al release upon the release and reaction of Biosilicate^®^-derived Ca.

The liberation of fluoride ions is a key advantage of GICs, as previously stated [8,9]. Fluoride enhances the acid resistance of teeth by inhibiting demineralization and promoting remineralization. Additionally, it inhibits bacterial growth and metabolism by suppressing the activities of enzymes such as enolase and ATPase [11,12]. The incorporation of Biosilicate^®^ resulted in a 4-fold increase in fluoride release for Maxxion R and a 2.5-fold increase for Fuji IX, as shown in Figure 7F,L. The addition of Biosilicate^®^ to both GICs resulted in a proportional increase in therapeutic ion release. This indicates that these materials have the potential to remineralize both enamel and dentin.

Incorporating a biological aspect into exploratory research is worthwhile. Multiple assays, including cytotoxicity, cell viability, attachment, morphology, migration, and differentiation, can facilitate the attainment of this objective. This study evaluated biocompatibility via an in vitro cytotoxicity assessment. The third hypothesis regarding the enhancement of biocompatibility by Biosilicate^®^ was rejected. No significant differences in cytotoxicity were found among the Fuji IX groups. However, the rate of cell death was significantly higher in M5% and M10% compared to M0%. The high alkalinity of Biosilicate^®^ in Fuji IX may account for this, as the glass ceramic appears to neutralize the acidity in these groups with a 1.8/1 ratio (g/g), resulting in a similarity to F0% (ratio 3.6/1 (wt/wt)). The minimal release of Al ions from Fuji IX groups may contribute to their non-cytotoxic nature.

The Maxxion R groups exhibited a significant release of ions, specifically Al and Na, in contrast to the other groups. M5% demonstrated the highest cytotoxicity, possibly due to its release of 105 ppm of Al. The increase in Al concentration (4.5 ppm, 45 ppm, and 450 ppm) is associated with a decrease in the percentage of live cells and an increase in the percentage of early apoptotic cells, late apoptotic cells, and dead cells [60]. Aluminum toxicity primarily induces oxidative stress through the overproduction of reactive oxygen species (ROS) [61], which disrupt signaling processes, inhibit cell growth, promote DNA damage [62,63], destroy phospholipidic membranes, and induce cellular apoptosis [64].

The fourth research hypothesis, that a higher amount of Biosilicate^®^ would lead to greater inhibition of *S. mutans*, was also rejected. Figure 9A clearly demonstrates that only M5% consistently killed *S. mutans* to levels below our limit of detection (less than 100 CFU/mL). M0% and M10% also significantly killed *S. mutans* compared to the no-disk control (N), but this killing was not significantly different from that of M15%. M5% released more Al (105 ppm) and may have contributed to the antimicrobial killing observed in the direct contact assay due to a higher amount of free Al on its surfaces. Aluminum has been observed to exhibit synergistic properties with fluoride, resulting in an increased bactericidal effect on *S. mutans* by inhibiting ATPase [65]. The possible mechanism of the combined action of fluoride and aluminum in inhibiting ATPase may involve the formation of an ADP-Al-F_3_ complex in the enzyme’s catalytic site [66,67]. ATPase plays an important role in the maintenance of the intracellular pH by pumping out protons; the inhibition of this enzyme disrupts the bacterial metabolism and the aciduric capability of *S. mutans* [68].

A significant reduction in *S. mutans* (NCTC 10449) growth after exposure to F^−^, Al^3+^, and SiO_3_^2−^, at respective concentrations of 8.6 ppm, 6.8 ppm, and 9.7 ppm has been reported previously [69]. In the same study, it was demonstrated that, as the concentration of all ions increased, the inhibitory effect tended to increase. In this study, M5% released 30 ppm of F^−^, 105 ppm of Al^3+^, and 19 ppm of SiO_3_^2−^. The synergistic effect of these ions may have enhanced the bactericidal activity against *S. mutans*. Al is the primary contributor to the antimicrobial effect observed on the surface of M5%, with F and Si playing a minor role.

Lastly, this was a prospection and comprehension study designed to better comprehend the potential benefits of the interaction between Biosilicate^®^ and two distinct GICs. Insufficient information was provided by the manufacturer regarding the composition and quantity of elements in the matrix, making it difficult to understand the interaction between the elements studied. The absence of compositional data required a surface analysis using energy-dispersive X-ray spectroscopy (EDX).

Further research is necessary to thoroughly explore the topic of improving the ion-releasing and antimicrobial properties of GICs, despite the promising advancements discussed in this article. This will require the use of different glass and ceramic compositions. The study proposes various methods for enhancing the properties of GIC, including altering their composition by increasing aluminum and decreasing calcium and sodium, testing different particle sizes of Biosilicate^®^, and employing alternative methods for incorporating glass ceramic into GIC, such as using a molar ratio instead of %wt, automated methods for better homogenization of powders, and different formulations of polyacid with co-polymers instead of PAA.

## 5. Conclusions

Incorporating Biosilicate^®^ at a weight percentage of 5% (wt) did not significantly alter the setting and mechanical properties of Maxxion R in this study. However, it did enhance ion release and antimicrobial activity. The addition of Biosilicate^®^ did not alter the cytotoxicity of Fuji IX, but it led to an increase in the release of therapeutic ions such as Si, P, Na, and Ca. Fuji IX’s mechanical properties and setting behavior were affected by incorporating Biosilicate^®^.

## Figures and Tables

**Table 1:** Manufacturers and composition of the materials used.

Material (Manufacturers)	Composition
Biosilicate^®^ (LaMaV-CeRTEV, São Carlos, SP, Brazil)	23.75% Na_2_O, 23.75% CaO, 48.5% SiO_2_, and 4% P_2_O_5_ (%wt), microparticles (D_50_ = 5 μm).
Maxxion R, Shade A2 (FGM, Joinville, Santa Catarina, Brazil)	Powder: aluminofluorosilicate glass and calcium fluoride (10 g). Liquid: polycarboxylic acid, tartaric acid, and water (8 g).
Fuji IX GP, Shade A2 (GC Corporation, Tokyo, Japan—authorized by South America GC, SP, Brazil)	Powder: aluminofluorosilicate glass and polyacrilic acid. Liquid (4 mL): polyacrylic acid and water.

**Table 2:** Groups of experimental formulations by % wt and P/L ratios.

Groups	Formulations (wt%)	Ratio P/L (wt/wt)
M0%	100% Maxxion R	1.5/1
M5%	95% Maxxion R + 5% Biosilicate^®^	1.5/1
M10%	90% Maxxion R + 10% Biosilicate^®^	1.5/1
M15%	85% Maxxion R + 15% Biosilicate^®^	1.5/1
F0%	100% Fuji IX	3.6/1
F5%	95% Fuji IX + 5% Biosilicate^®^	1.8/1
F10%	90% Fuji IX + 10% Biosilicate^®^	1.8/1
F15%	85% Fuji IX + 15% Biosilicate^®^	1.8/1

## Data Availability

Not applicable.
